# Calorimetric Method for the Testing of Thermal Coefficients of the TIG Process

**DOI:** 10.3390/ma15207389

**Published:** 2022-10-21

**Authors:** Marek Mróz, Antoni Władysław Orłowicz, Magdalena Lenik, Andrzej Trytek, Mirosław Tupaj

**Affiliations:** 1 Department of Foundry and Welding, Rzeszow University of Technology, Powstancow Warszawy 12, 35-959 Rzeszow, Poland; 2 Faculty of Mechanics and Technology, Rzeszow University of Technology, Kwiatkowskiego 4, 37-450 Stalowa Wola, Poland

**Keywords:** TIG method, aluminium–silicon alloys, calorimeter, arc efficiency η, melting efficiency η_m_

## Abstract

This paper presents an original design of a test apparatus for calorimetric measurements of arc efficiency η and melting efficiency η_m_ in welding processes. The construction and principle of operation of a new flow calorimeter are described, as well as the method for determining the η and η_m_ values in the process of the surface melting of aluminium–silicon alloy casting surfaces with a concentrated heat flux generated by the TIG (Tungsten Inert Gas) method. The results obtained indicate the advisability of using calorimetric testing to assess the arc efficiency of welding processes. It was demonstrated that changing the welding current and arc scanning speed, as well as changing the chemical composition of the silumin, has an effect on the arc efficiency value η. This has the effect of introducing a different amount of heat into the area of the heated material. The consequence of this is a change in the value of the melting efficiency η_m_, which results in a change in the width and depth of the surface melting areas, through this, the cooling conditions of the material. As is well known, this will affect the microstructure of the welds and the width and microstructure of the heat-affected zone, and thus the performance of the welded joints.

## 1. Introduction

The performance of welded joints depends on the chemical composition and microstructure of the base material, the geometry and microstructure of the welds and the width and microstructure of the heat-affected zone (HAZ). The weld geometry and the width of the heat-affected zone are determined by the amount of heat introduced into the joint during the welding process. The amount of heat introduced has an effect on the primary crystallisation of the welds and the course of phase transitions in the heat-affected zone during the cooling of the welded joints [1,2,3].

As the microstructure of the alloys used in the different types of structures determines their mechanical properties and fatigue strength, it is important to know how it can be modelled in the welded joints in the weld and heat-affected zone areas. The welding process parameters used in the manufacturing of structures should be such that, with the set cooling conditions, enough heat can be introduced into the welded joint to ensure that, in the weld and the HAZ, a microstructure is formed, which provides levels of mechanical properties, and a fatigue strength not inferior to those of the base material [3,4,5].

Consequently, the development of the welding procedure for the respective structures includes the study of the influence of the welding process parameter values on the amount of heat introduced into the welded joint and its effect on the weld geometry and the width of the HAZ, the determination of the type of microstructure components formed, their volume proportions and material property values, followed by the study of the mechanical properties and fatigue strength. Their results are the basis for the development of empirical relationships linking the amount of heat introduced to the weld geometry and its microstructure, the width of the HAZ and its microstructure, and the mechanical properties and fatigue strength. It is sometimes necessary to carry out such tests for cooling conditions with isothermal endurance, for example, when there is a need to block the martensitic transformation in the weld and HAZ [6,7].

Such studies constitute the know-how of leading responsible construction manufacturers and are not usually published. Sometimes these companies agree to publish partial research results or to patent them.

Since the amount of heat introduced into a welded joint depends on many factors— for example, the chemical composition of the parts to be joined and their microstructure, the current waveforms obtained from the current sources, the set values of the current parameters, the length of the arc, the speed of the arc scanning, and the protective atmosphere used—the precise determination of the amount of heat introduced into the heated material requires the use of experimental methods. The results of such tests allow the value of an important parameter of the electric arc welding process to be calculated, namely the arc efficiency η. The arc efficiency of the welding process indicates how much of the energy supplied to the heated material was used to form a surface melting area or weld [8].

**Figure 1:**
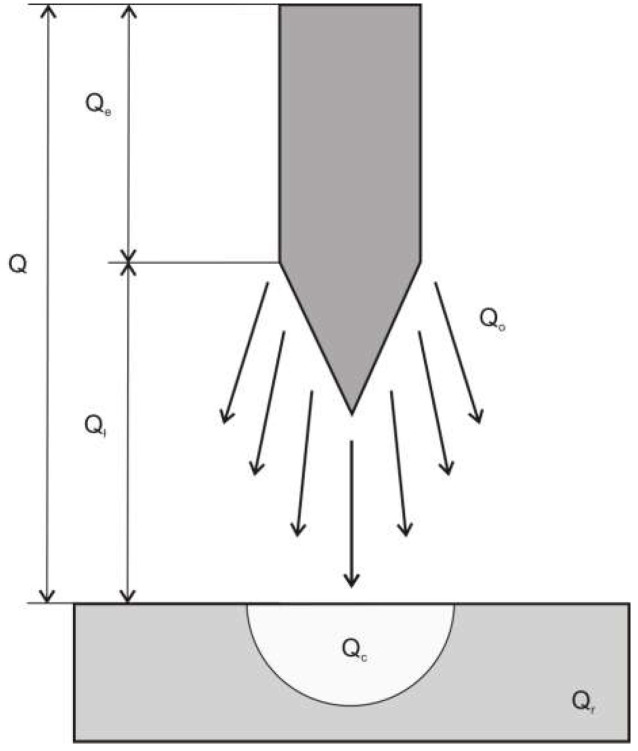
Energy distribution in the TIG process: Q, total heat generated in the electric arc Q_arc_ and on the electrode Q_e_; Q_o_, amount of heat transferred to the surrounding area; Q_c_, amount of heat required to heat and partially melt the material and then overheat the liquid metal; Q_r_ + Q_c_ is that part of the heat absorbed by the heated element; Q_r_, amount of heat transferred by conduction to the parent material.

To clarify the issue of heat generation and heat flows in TIG (Tungsten Inert Gas) welding, Du Pont and Marder [9] proposed the heat balance diagram shown in Figure 1.

The heat balance of the process shown in Figure 1 can be expressed by the equation:

(1) $$Q=Q_{\mathrm{arc}}+Q_{e}+Q_{o}+Q_{c}+Q_{r,}$$

where Q is the total heat generated in the welding process (J), Q_arc_ is the amount of heat generated in the electric arc (J), Q_e_ is the amount of heat generated in the electrode (J), Q_o_ is the amount of heat transferred to the surrounding area (J), Q_c_ is the amount of heat needed to heat and melt the metal and further overheat the liquid metal (J), and Q_r_ is the amount of heat transferred by conduction to the parent material (J).

The main amount of heat is generated in the arc (Q_arc_), and only a small amount (Q_e_) is generated in the electrode. Part of the heat (Q_o_) goes directly into the surrounding space, and the remainder (Q_r_ + Q_c_) penetrates the heated material. Heat (Q_c_) is used to form a pool of liquid metal and overheat the liquid within it. Heat (Q_r_) diffuses in a direction from the liquid metal pool into the parent material, heating it up. In the area heated to temperatures where phase transformations take place, microstructural changes occur. This area is called the heat-affected zone (HAZ). The width of this zone increases with an increase in the time of the spot heat effect of the electric arc.

For the calculation of the theoretical amount of heat Q_teor_ generated in an electric arc, the following expression is used:

(2) $$Q_{\mathrm{teor}}=Q_{\mathrm{arc}}=U\cdot I\cdot t$$

where U is the arc voltage (V), I is the welding amperage (A), and t is the scanning time (t).

The degree to which the heat released in the electric arc is used to heat the material is characterised by the arc efficiency of the process η. The arc efficiency of the process is described by an expression involving the quotient of the measured amount of heat taken up by the heated sample Q_cal_ = Q_r_ + Q_c_ and the theoretical amount of heat generated in the electric arc Q_teor_:

(3) $$\eta =\frac{Q_{\mathrm{cal}}}{Q_{\mathrm{teor}}}=\frac{Q_{c}+Q_{r}}{Q_{\mathrm{teor}}}$$

The degree of utilisation of the heat absorbed by the material to produce surface melting is characterised by the melting efficiency η_m_. The melting efficiency is described by the following expression:

(4) $$\eta =\frac{Q_{c}}{Q_{c}+Q_{r}}$$

From the expressions presented, it can be seen that the selection of parameters for the surface melting process should be carried out in such a way as to maximise the value of the arc efficiency of the process and the melting efficiency. It should be carried out in such a way as to limit energy losses and minimise the width of the heat-affected zone.

The arc efficiency η of the TIG process is calculated from the following expression:

(5) $$\eta =\frac{Q_{\mathrm{cal}}}{U\cdot I\cdot t}$$

where Q_cal_ is the amount of heat absorbed by the heated material, determined experimentally using a calorimeter (J), U is the arc voltage (V), I is the amperage (A), and t is the arc scanning time (t).

The melting efficiency is calculated from the following expression:

(6) $${\;\eta }_{m}=\frac{V_{n}\cdot \rho \cdot Q_{H}}{Q_{\mathrm{cal}}}$$

(7) $$Q_{H}={\Delta Q}_{f}+\int_{T_{0}}^{T_{1}}c_{p}\mathrm{dT}$$

where V_n_ is the surface melting area volume (mm^3^), *ρ* is the alloy density (g/mm^3^), Q_H_ is the heat required to heat a unit volume of the alloy from the temperature T_0_ to the temperature T_1_ and melt it (J/g), Q_f_ is the melting heat (J/g), and c_p_ is the specific heat (J/gK).

The main problem in calculating arc efficiency is determining the amount of heat taken up by the heated element.

Two lines of research on this issue can be distinguished in the literature. The first, using methods of mathematical analysis of the heat transfer process, involves searching for the temperature distribution in the material obtained as a result of the action of concentrated heat flux. The temperature field equations presented in the literature, based on a heat source model and a heat flux model founded on a Gaussian distribution function, were obtained by assuming that the values of the material constants of the heated workpieces are invariant with temperature. This approximation distorts the true picture of the phenomena occurring between the arc and the scanned surface. Taking into account the variation of these properties as a function of temperature complicates the calculation flow considerably. Progress on this issue has been made through the use of numerical methods. The application of these methods to describe the heat conduction process during the arc plasma scanning of the workpiece surface has been addressed by a number of researchers [10,11,12,13,14]. Numerical methods allow complex and lengthy calculations, which in turn make it possible to take into account the effect of temperature on the change in the physical properties of the heated material and to analyse the phenomenon as a sum of instantaneous states. Thermal arc efficiency values, calculated in this way, are in the range of 75–80%. They raise doubts primarily concerning two issues. Firstly, there is insufficient experimental verification of them. Secondly, the adopted values of the conductivity coefficient of the liquid metal (pool) are up to four times higher than the value of this coefficient at the melting temperature. It would, therefore, appear that the arc efficiency values quoted are an overestimate for results close to the experimental results. In summary, it can be said that in studies on the amount of heat applied to the superheated surface, the results obtained, depending on the assumptions made, are more or less close to reality and often differ [15].

Therefore, the final results of this modelling should be treated with due caution. For this reason, the results of calculating the heat introduced into the heated material must be verified by experimental methods [16].

**Figure 2:**
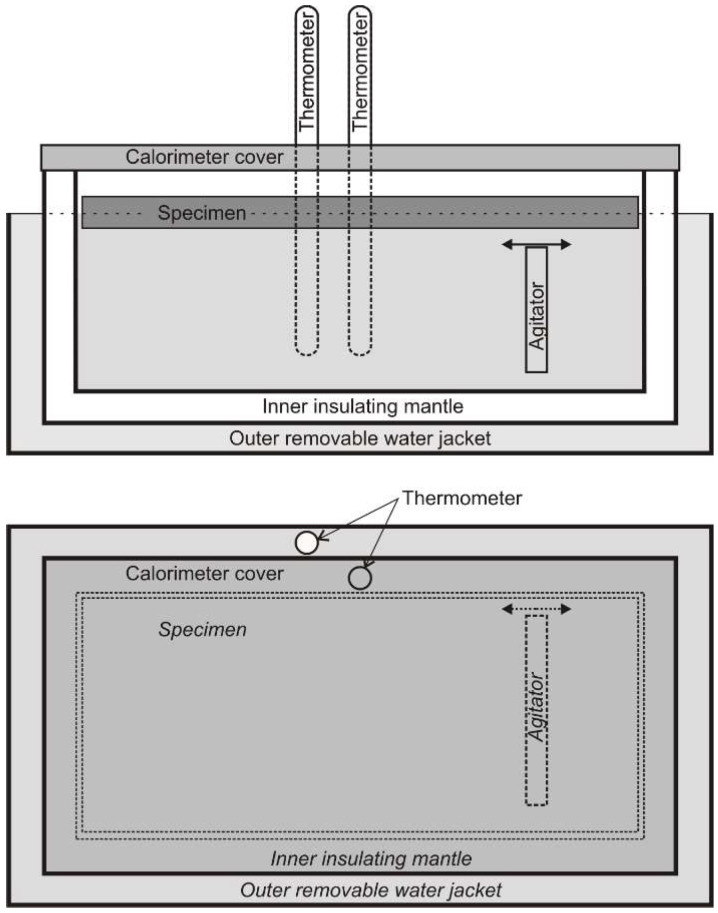
Havaldas’s calorimeter [18].

This second line of research is based on calorimetric measurements of the amount of heat taken up by the superficially melted workpiece [10,15,16,17,18,19,20,21,22,23]. There are few studies in the technical literature on calorimetric measurements of welding processes. Various measurement techniques are used for this purpose. For example, Havalda [18] placed the sample to be hardfaced above the surface of the water filling the calorimetric vessel (Figure 2).

The appropriate design of the holder allowed it to be quickly submerged in water after the bead was made. Once the sample was immersed, the vessel was closed with a lid. Based on the predetermined water constant of the calorimeter (the amount of heat that corresponds to a 1 °C increase in the temperature of the water in the calorimetric vessel) and on measurements of the increase in water temperature, the amount of heat introduced into the calorimeter was determined. The measurement technique used required taking into account the amount of heat used to evaporate the water as a result of the electric arc and during the immersion of the sample.

**Figure 3:**
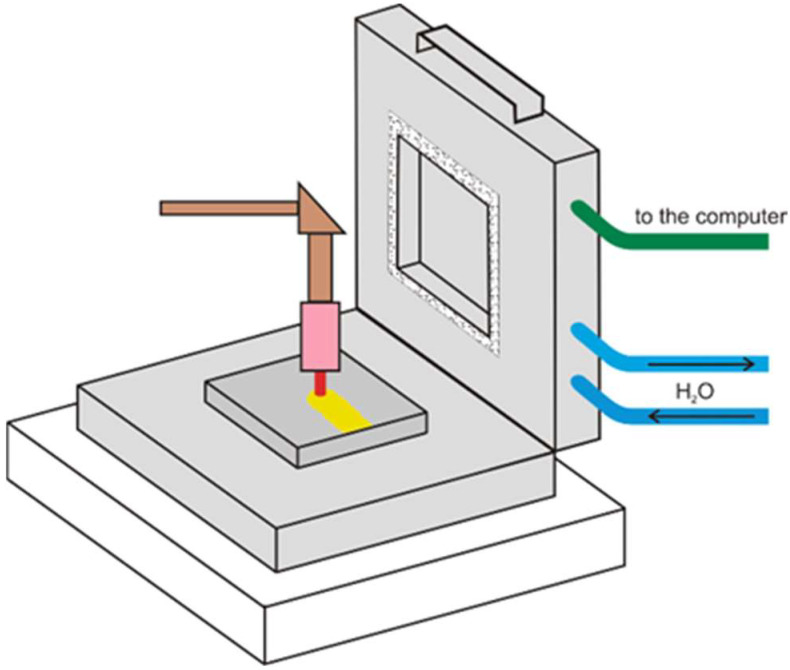
The Seebeck calorimeter [8].

Giedt, Knorowski and other authors [8,22] determined the arc efficiency of the TIG process using a Seebeck calorimeter (Figure 3).

With the calorimeter lid open, a melting section was performed on the sample placed on the base of the calorimeter. The lid was then closed. The correct orientation of the heat flow in the lid was enforced by the flow of water in the cooling pipes. The temperature drop across the walls of the calorimeter was also assessed and measured using a set of thermocouples.

Heat loss due to radiation and convection was estimated to be about 1% of the energy supplied by the arc. The inconvenience of the calorimetric measurements with a Seebeck calorimeter is the long measurement cycle time, reaching six hours in some cases.

**Figure 4:**
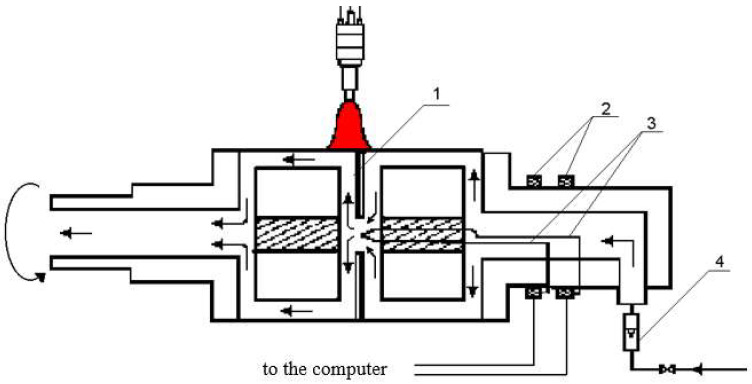
Flow calorimeter diagram; 1, gasket; 2, thermocouple tip sliding contacts; 3, thermocouple; 4, flow meter [19].

Another design of apparatus for calorimetric measurements in welding processes is the Krzyzanowski calorimeter [19] in the form of a rotating cylinder made of brass sheet (Figure 4). During measurements, the outer surface of the cylinder is scanned with concentrated heat flux. The amount of heat input is assessed by measuring the flow rate of water through the cylinder and the difference in temperature of the water at the calorimeter’s outlet and inlet lines.

The Havalda calorimeter and the Seebeck calorimeter present some disadvantages that affect the measurement results. First, the sample is superficially melted and then immersed in water. This involves heat loss before the actual measurement. The Seebeck calorimeter has the problem of unrecorded heat flow into the body on which the superficially melted sample rests. In the Krzyzanowski calorimeter, the samples would have to be tube-shaped. The calorimeter presented in this study does not suffer from such disadvantages.

**Figure 5:**
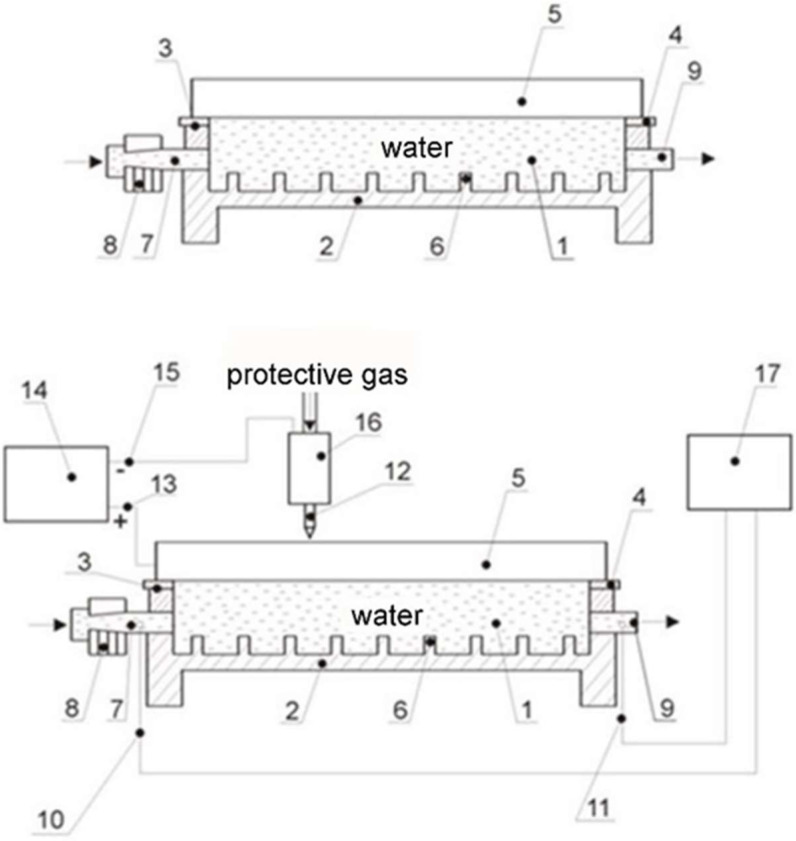
Structural design of the flow calorimeter; 1, water; 2, flow chamber; 3, top edge of the chamber; 4, gasket; 5, superficially melted sample; 6, partitions; 7, water inlet pipe; 8, flow rotameter; 9, water drainage pipe; 10, 11, thermocouples; 12, tungsten electrode; 13, positive pole; 14, power source; 15, negative pole; 16, holder with protective gas; 17, temperature recording [23].

A new design of flow calorimeter for measuring thermal quantities characterising welding processes is the subject of Polish patent no. 211283 [23]. The flow calorimeter takes the form of a rectangular vessel. The design of the flow calorimeter is shown in Figure 5.

The sample is placed at the top. Water flowing through the calorimeter rinses the lower surface of the sample. The water flow rate on the flow rotameter is chosen so that neither turbulence nor gas bubbles appear at the sample–water interface. Water is supplied via a slotted inlet, and the water temperature is measured in both the inlet and outlet pipes. The actual measurements start once the temperature of the outflowing water has stabilised, which usually occurs a few seconds following the start of the melting of the sample surface.

**Figure 6:**
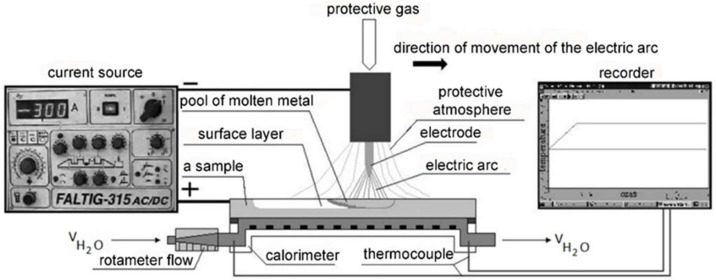
Diagram of the flow calorimeter test system.

A diagram of the test system using the flow calorimeter is shown in Figure 6.

The aim of this study was to present the measurement capabilities and test methodology using the new calorimeter and to present example results of tests of the effects of TIG welding current and arc scanning speed on the arc efficiency of the process, the melting efficiency and the geometry of the surface melting areas of AK 51 and AK 20 alloy samples.

## 2. Materials and Methods

### 2.1. Material and Casting Procedure

The test material consisted of die-cast plates measuring 260 × 55 × 15 mm made from an aluminium–silicon alloy. AK 51 and AK 20 alloys were used in the study. The chemical composition of the alloys is given in Table 1.

Smelting was carried out in a 300 kg electric resistance furnace. In the case of the AK 51 alloy, the metal feedstock was scrap metal from the AK 51 alloy castings. A refining treatment was carried out at 710 °C with Dursalit EG 281 at 500 g per 100 kg of metal. Five minutes after the refining procedure and the removal of oxides from the liquid metal surface, the metal moulds of the test plates were flooded with the alloy at 710 °C. The AK 20 alloy was prepared on the basis of the AK 20 pre-alloy. Refining was carried out at 740 °C with Dursalit EG 281 at 500 g per 100 kg of metal. Five minutes into the treatment, the metal moulds of the test plates were flooded with the alloy at 750 °C.

The chemical composition of the calorimetric samples was analysed using a Q4 Tasman Bruker spectrometer, Bruker Poland, Poznań.

### 2.2. Arc and Melting Efficiency

**Figure 7:**
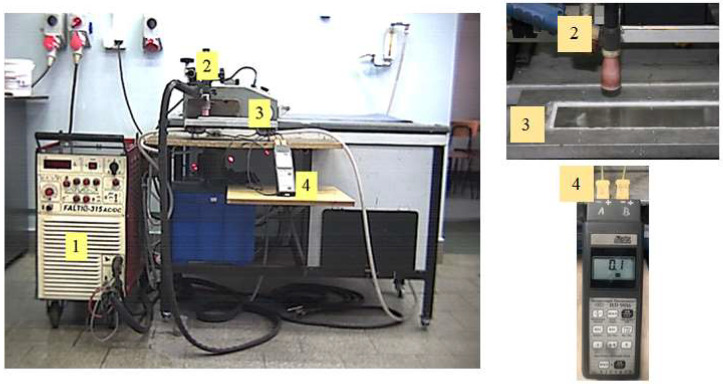
Calorimetric test bench; 1-TIG welding machine, 2—welding torch with tungsten electrode, 3—flow calorimeter with sample, 4—temperature recorder.

After mounting in the flow calorimeter, the plate casts were superficially melted with electric arc plasma using a Faltig 315 AC/DC device, Ozas company, Opole, Poland. The test bench is shown in Figure 7. A 4 mm diameter tungsten electrode was used. The surface melting was performed in an argon atmosphere. The shielding gas output was 20 l/min. Amperage levels of I = 100, 150, 200, 250 and 300 A and arc scanning speeds of vs. = 200 and 800 mm/min were used.

The calorimeter was in the shape of a cuboidal closed tub (Figure 5) through which water flowed. The water was supplied via a slotted inlet. The water temperature was measured at the inlet and outlet pipes. From the top, the calorimeter was covered with a superficially melted sample. The water flowing through the calorimeter rinsed its lower surface. With a water outflow of 7.5 L/min, no turbulence or vapour bubbles were observed during the surface melting cycle of the samples. The water temperature stabilised after 10 s. Calorimetric measurements were made for 20 cm long surface melting areas.

Each measurement point was developed for three non-overlapping beads made on the same sample plate. The automated travel of the welding torch ensured a repeatable scanning speed. Precise welding current settings were used. Precision control of the water flow in the calorimeter and computerised recording of water temperature values were used. This preparation of the test material and the conduct of the experiment ensured almost 100% reproducibility of the test results.

The amount of heat released in the arc Q_teor_ was determined from expression (2).

The amount of heat absorbed by the heated sample Q_cal_ was determined from the following relationship:

(8) $$Q_{\mathrm{cal}}=m_{w}\cdot c_{\mathrm{pw}}\cdot {\Delta T}_{w}$$

where Q_cal_ is the amount of heat absorbed by the calorimeter, m_w_ is the water mass, c_pw_ is the specific heat of water in the temperature range under investigation, and ΔT_w_ is the water temperature difference: ${\Delta T}_{w}=T_{w_{k}}-T_{w_{p}}$, $T_{w_{p}}$ is the initial water temperature, $T_{w_{k}}$ is the final water temperature.

The melting efficiency was calculated from relation (6).

### 2.3. Fusion Geometry Examination

Measurements of the width and depth of the surface melting areas were taken using a Nephot 2 optical microscope, Carl Zeiss, Jena, Germany. The cross-sectional area of the surface melting areas in a plane perpendicular to their longitudinal axis was assessed using a planimetric method based on their photographs. The results of these measurements were the arithmetic average of the ten analysed welding cross-sections perpendicular to their longitudinal axis.

### 2.4. Microstructure Parameters

Due to the strong fragmentation of the microstructure in the surface melting areas, the value of the eutectic structural parameter λ_E_ was assessed on images of the microstructures taken with a scanning microscope at sufficiently high magnifications.

## 3. Results and discussion

### 3.1. Arc efficiency and melting efficiency

The results of calorimetric tests of the surface melting process of AK 51 and AK 20 alloy castings are shown in Table 2 and Table 3.

The results of the arc efficiency tests of the process of TIG melting indicate that, for both materials tested, the values of this coefficient increase with increasing welding current and decreasing arc scanning speed. This results in a similar nature of the effect of welding parameter values on the amount of heat introduced into both test materials.

It turned out that alloy AK 20 had slightly higher arc efficiency values and significantly higher melting efficiency values. This suggests its greater susceptibility to forming surface melting areas.

**Figure 8:**
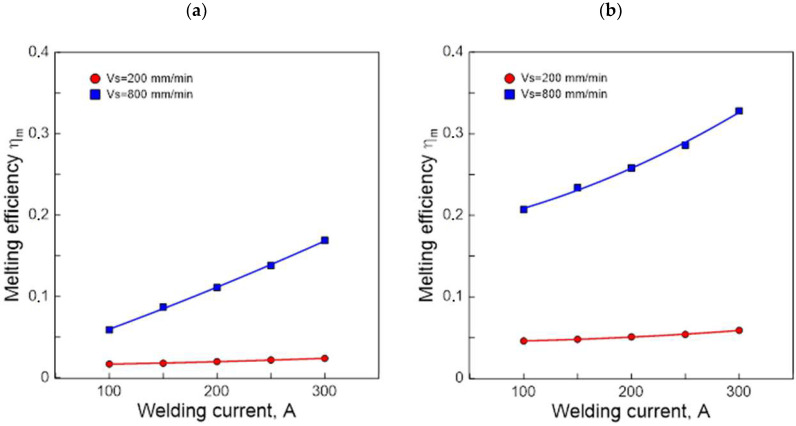
Effect of welding current and scanning speed of TIG arc welding in an argon atmosphere on melting efficiency in calorimetric testing of AK 51 (**a**) and AK 20 (**b**) alloys samples.

Figure 8 illustrates graphically the test results (nature) of the effect of welding current and arc scanning speed on the TIG melting efficiency of the AK 51 alloy and AK 20 alloy samples.

The results show that higher values of arc efficiency and melting efficiency were obtained for higher welding current and lower arc scanning speeds. Alloy AK 20 was found to have a higher susceptibility to the formation of surface melting areas.

The results of the tests indicate that, due to the achievement of surface melting areas with a large depth and width, the selection of the values of the TIG process parameters should be carried out in such a way as to maximise the value of the arc efficiency of the welding process.

On the other hand, it is known that low-depth low-width surface melting areas have a lower heat capacity compared to high-depth, high-width surface melting areas. Consequently, their cooling rate is higher, resulting in a more strongly fragmented microstructure. It is known that in the case of silumins, the fragmentation of the microstructure provides higher levels of mechanical properties and fatigue strength.

The importance of the arc efficiency value of the welding process in scientific work on developing relationships between the amount of heat input and the performance of welded joints is not always appreciated, as shown in study [24]. In calculating the amount of heat introduced into the welded joints of aluminium alloy sheets, it adopted one and the same efficiency value from the literature, η = 0.8, although the welding current values varied from 135 to 180 A and the linear welding speed from 5.9 to 7.32 mm/s.

Du Pont and Marder [9], Smartt et al. [23] and Giedt et al. [8] determined the thermal efficiency of TIG welding steel. For the range of the welding current values used, similar to those presented in this study, the thermal efficiencies were 0.67, 0.7 and 0.8.

Fuerschbach and Knorowsky [22] determined a thermal efficiency of 0.3.

The flow calorimeter used in the present paper was used in study [25], in which surface melting was performed on the surface of AZ 91 alloy castings using the TIG method. The amperage varied from 100 to 400 A, and the welding speed from 200 to 400 mm/min. The authors found that an increase in arc scanning speed is accompanied by a decrease in the arc efficiency of the process, while an increase in welding current increases the arc efficiency value of the process. The range of variation in thermal efficiency values was 0.63–0.88. The authors of paper [23] state that the effect of an increase in the arc efficiency of the process is an increase in the amount of heat introduced into the surface melting areas, thus increasing its width and depth. The amount of heat introduced into the surface melting areas influences the conditions for their crystallisation through which their microstructure is determined.

It has also been used to evaluate the geometry of the surface melting areas and the arc efficiency of the surface melting process of aluminium–silicon alloy [26,27,28], cast iron [29,30,31], and cobalt alloy castings [32,33].

### 3.2. Fusion Geometry

The results of measuring the width and depth of the surface melting areas and the results of calculating the cross-sectional area and volume of the surface melting areas on the AK 51 and AK 20 alloy samples are shown in Table 4 and Table 5.

The results of the melt geometry tests indicate that for both materials tested, the depth and width of the surface melting areas increase with increasing welding current and decreasing arc scanning speed, with the width of the surface melting areas being greater than their depth. This results in a similar nature to the effect of welding parameter values on the cross-sectional area of the surface melting areas and their volume assessed over the same length of 20 cm.

**Figure 9:**
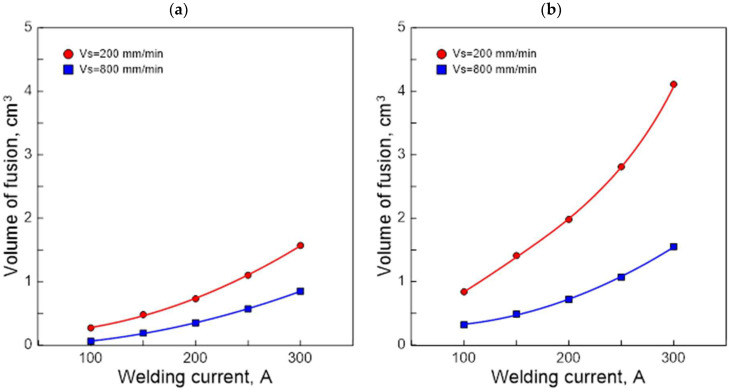
Effect of welding current and arc scanning speed values using the TIG method in an argon atmosphere on the volume of 200 mm long surface melting obtained from calorimetric measurements of AK 51 (**a**) and AK 20 (**b**) alloys samples.

The AK 20 alloy was found to be more prone to surface melting. That is, at the same welding current and arc scanning rate, the width and depth of surface melting on the AK 20 alloy samples were greater than on the AK 51 alloy samples. This results in higher values for the volume of surface melting obtained in the case of alloy AK 20. Figure 9 shows the results (nature) of the effect of welding current and arc scanning speed on the volume of 200 mm of surface melting for the AK 51 and AK 20 alloy samples.

### 3.3. Microstructure

**Figure 10:**
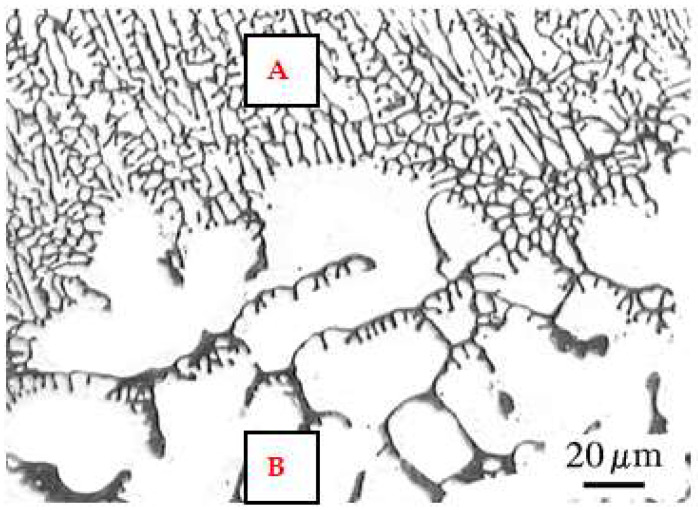
Microstructure of the AK 51 alloy in the surface melting area (top—area A) and in the native material area (bottom—area B). In the surface melting area, there is eutectic α(Al) + β(Si) and very fine dendrites of the α(Al) phase. Etch with Dix and Keith reagent.

**Figure 11:**
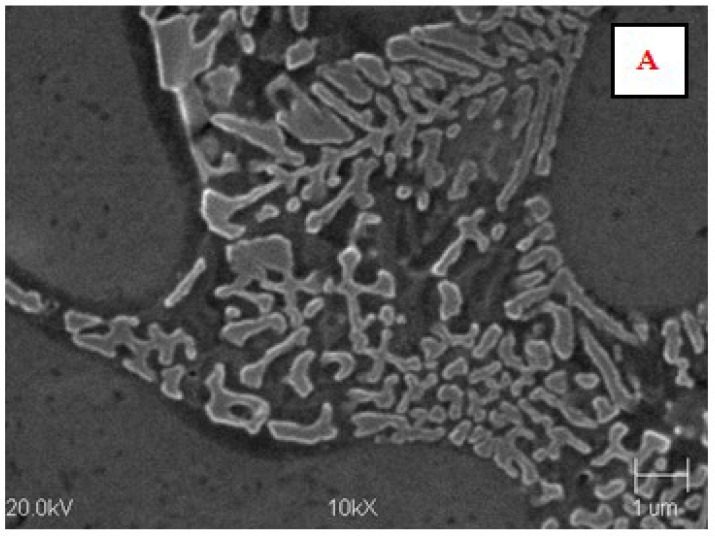
Microstructure of AK 51 alloy surface melting using the TIG method in an argon atmosphere at a welding current of I = 150 A and a scanning speed of vs. = 800 mm/min. A is the area in Figure 10. An eutectic characterised by a distance between lamellae and silica fibres λ_E_ of less than 1 µm. Etch with Dix and Keith reagent.

**Figure 12:**
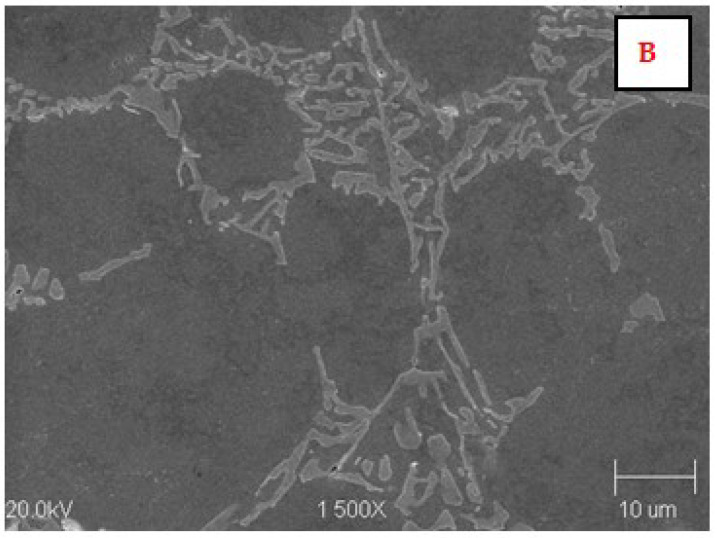
Microstructure of the AK 51 alloy in the native material area. B is the area in Figure 10. Visible lamellar and fibrous eutectics with the value of the parameter λ_E_ on the order of a few micrometers and the dendrites of α(Al) phase. Etch with Dix and Keith reagent.

**Figure 13:**
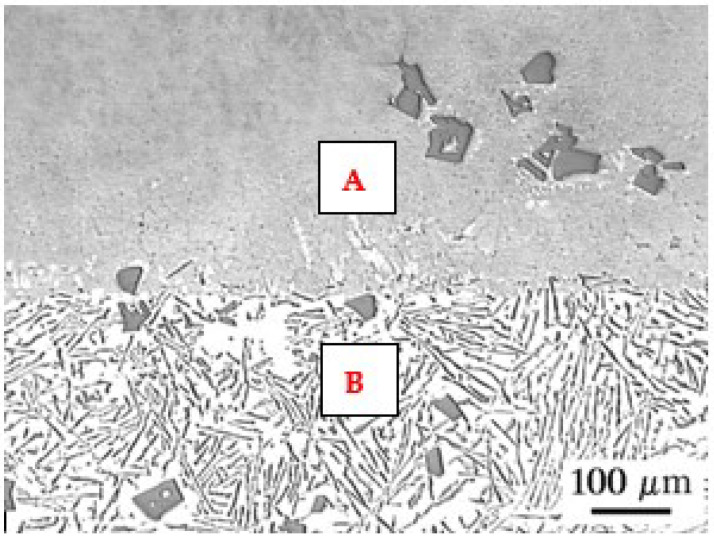
Microstructure of the AK 20 alloy in the surface melting area (top—area A) and in the native material area (bottom—area B). In the surface melting area, there is a eutectic, undissolved primary silicon crystals and very fine dendrites of the α(Al) phase at the super-melting boundary and around the primary silicon crystals. Etch with Dix and Keith reagent.

**Figure 14:**
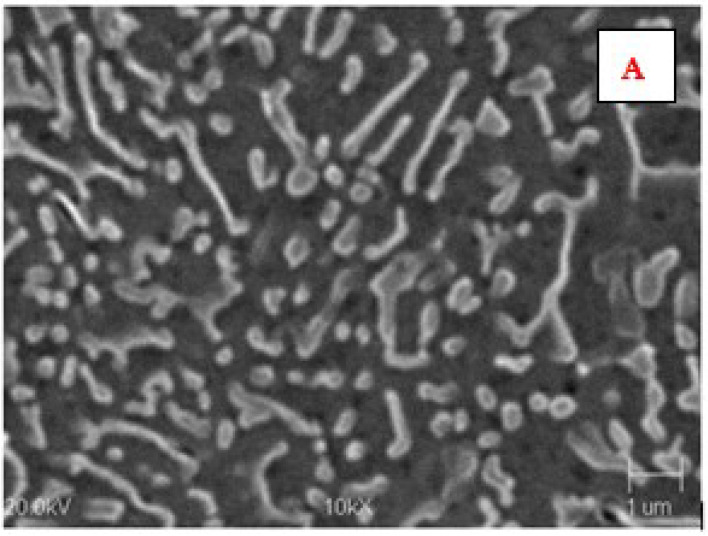
Microstructure of AK 20 alloy surface melting using the TIG method in an argon atmosphere at a welding current of I = 150 A and a scanning speed of vs. = 800 mm/min. A is the area in Figure 10. An eutectic characterised by a distance between lamellae and silica fibres λ_E_ of less than 1 µm. Etch with Dix and Keith reagent.

**Figure 15:**
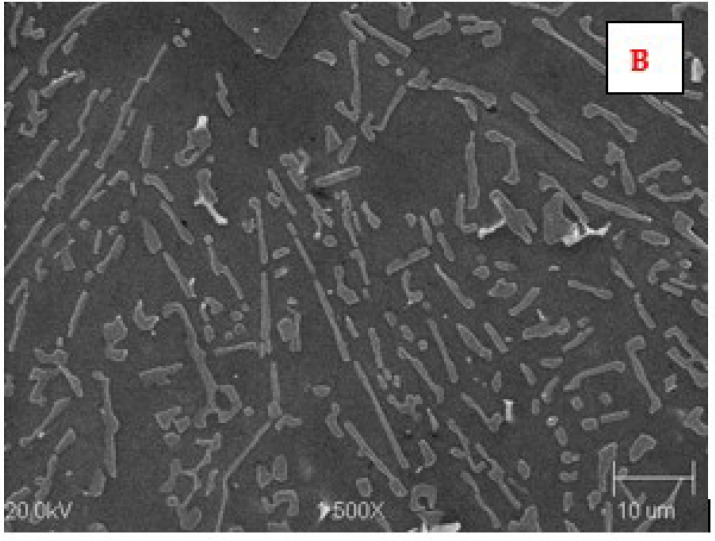
Microstructure of the AK 20 alloy in the native material area. B is the area in Figure 10. A visible lamellar and fibrous eutectic with an λ_E_-value of a few micrometres and a primary silicon crystal (top). Etch with Dix and Keith reagent.

The microstructure of the AK 51 and AK20 alloys in the areas adjacent to the fusion line on the surface melting side and on the native material side are shown in Figure 10, Figure 11, Figure 12, Figure 13, Figure 14 and Figure 15.

In the microstructure of the surface melting area, made on castings of AK 51 alloy, there is eutectic α(Al) + β(Si) and very fine dendrites of the α(Al) phase, compared to the native material.

Compared to the substrate material, the value of the structural parameter λ_E_ of the α + β eutectic is several times smaller. The λ_E_ parameter is very sensitive to the temperature gradient in the liquid metal pool [34,35]. The different values of this parameter in the case of the base material, which was a die casting, and in the case of surface melting are indicative of the different values of the temperature gradients in the liquid melt. Therefore, for surface melting differing in geometry, different values of temperature gradients are expected to be present, resulting in an eutectic with different values of the structural parameter λ_E_ during the crystallisation process. On the other hand, it is known that the geometry of surface melting areas depends on the flows of liquid metal in the welding pool as a result of electromagnetic interactions (Lorentz forces) and as a result of Marangoni convection [36].

Aluminium–silicon alloys with a structure containing thick silicon lamellae have a low level of mechanical properties. These properties improve as the shape of the Si precipitates changes from lamellar to fibrous. This can be achieved as a result of rapid crystallisation.

## 4. Conclusions

The results obtained indicate the suitability of the newly developed calorimeter for testing the arc efficiency of the welding process. Correct data on the arc and melting efficiency values allow the necessary amount of heat to be designed that needs to be introduced into the joint in order to achieve the assumed weld geometry and its microstructure, as well as the width of the HAZ and its microstructure.

The effect of the surface melting of Al-Si alloy castings using concentrated heat flux is the rapid crystallisation of the superficially melted area. On the other hand, the effect of rapid crystallisation is a highly fragmented microstructure (low value of the λ_E_ parameter). The consequence will be an increase in performance.

Variations in amperage and arc scanning speed affect arc efficiency, melting efficiency and the amount of heat introduced into the heated material. This provides the opportunity to control the geometry of surface melting areas. In turn, changes in temperature gradients and the cooling conditions of the liquid metal pool affect the structure of the surface melting area.

The thermal efficiency of the surface melting process decreases as the arc scanning speed increases and the welding current decreases. The highest arc efficiency values are obtained when low arc scanning speeds and high welding current values are used.

The effect of the microstructure of the base material on the value of arc efficiency and melting efficiency was found. This is related to the amount of heat introduced into the heated material, which affects the geometry of the surface melting areas. In turn, the amount of heat introduced into the heated material depends on the presence in the microstructure of varying volume proportions of its individual components and their morphology, which differ in the value of the thermal conductivity coefficient and the value of the temperature and heat of fusion.

## Figures and Tables

**Table 1:** Chemical composition of the alloy.

Alloy	Elemental Content, % by Weight							
	Si	Mg	Mn	Cu	Fe	Ti	Cr	Al
AK 51	5.56	0.38	0.34	0.24	0.26	0.110	0.009	remainder
AK 20	21.65	0.04	0.02	0.10	0.28	0.020	0.010	remainder

**Table 2:** Results of calorimetric tests of the surface melting process of AK 51 alloy plate castings.

No.	GTAW Process Parameters		Efficiency	
	I, A	v_s_, mm/min	arc, η	Melting, η_m_
1	100	200	0.74	0.017
2	150	200	0.77	0.018
3	200	200	0.80	0.020
4	250	200	0.82	0.022
5	300	200	0.84	0.024
6	100	800	0.56	0.059
7	150	800	0.58	0.087
8	200	800	0.60	0.111
9	250	800	0.61	0.138
10	300	800	0.62	0.169

**Table 3:** Results of calorimetric tests of the surface melting process of AK 20 alloy plate castings.

No.	GTAW Process Parameters		Efficiency	
	I, A	v_s_, mm/min	arc, η	Melting, η_m_
1	100	200	0.81	0.046
2	150	200	0.83	0.048
3	200	200	0.84	0.051
4	250	200	0.86	0.054
5	300	200	0.88	0.059
6	100	800	0.57	0.207
7	150	800	0.61	0.234
8	200	800	0.62	0.258
9	250	800	0.64	0.286
10	300	800	0.65	0.328

**Table 4:** Width a, depth h, cross-sectional area F and volume V of surface melting areas on AK 51 alloy samples.

No.	GTAW Process Parameters		Surface Melting Area Indicators			
	I, A	v_s_, mm/min	a, mm	h, mm	F, mm^2^	V_n_, cm^2^
1	100	200	3.54	0.43	1.37	0.27
2	150	200	5.05	0.69	2.62	0.48
3	200	200	6.57	0.96	4.31	0.73
4	250	200	8.09	1.21	6.34	1.10
5	300	200	9.57	1.45	8.62	1.57
6	100	800	1.04	0.19	0.26	0.06
7	150	800	2.48	0.45	0.98	0.19
8	200	800	3.94	0.69	2.06	0.35
9	250	800	5.24	0.92	3.35	0.57
10	300	800	6.45	1.11	4.73	0.85

**Table 5:** Width a, depth h, cross-sectional area F and volume V of surface melting areas on AK 20 alloy samples.

No.	GTAW Process Parameters		Surface Melting Area Indicators			
	I, A	vs, mm/min	a, mm	h, mm	F, mm^2^	Vn, cm^2^
1	100	200	5.63	2.10	7.26	0.84
2	150	200	6.47	2.65	10.24	1.41
3	200	200	7.28	3.25	13.86	1.98
4	250	200	8.17	3.82	17.99	2.81
5	300	200	8.97	4.38	22.38	4.11
6	100	800	3.17	1.37	2.97	0.32
7	150	800	3.88	1.90	4.79	0.49
8	200	800	4.55	2.49	7.13	0.72
9	250	800	5.36	2.99	9.80	1.07
10	300	800	6.25	3.57	13.32	1.55

## Data Availability

Not applicable.
